# Zhike Pingchuan Granule suppresses interleukin (IL)-6 or the medium of M2 macrophages induced apoptosis in human bronchial epithelial cells

**DOI:** 10.1080/21655979.2021.1982309

**Published:** 2021-10-05

**Authors:** Yumei Ren, Yongbin Yan, Lei Zhen, Caihong Cao, Quan Wang, Yingying Zhang, Shan Zhu

**Affiliations:** a Pediatric Department, The Second Clinical Medical College of Henan University of Traditional Chinese Medicine, Zhengzhou, China; b Pediatric Department, The First Affiliated Hospital of Henan University of Traditional Chinese Medicine, Zhengzhou, China; c Central laboratory, Central Laboratory, the Second Affiliated Hospital of Henan University of Traditional Chinese Medicine, Zhengzhou, China; d Basic Medicine, Henan University of Traditional Chinese Medicine, Zhengzhou, China

**Keywords:** Zhike Pingchuan Granule, IL-6, asthma, JAK2/STAT3

## Abstract

The aim of this study was to explore the effects and action mechanism of Zhike Pingchuan Granule in human bronchial epithelial cells induced by IL-6 or the supernatant of M2. Upon IL-6 stimulation at different doses, Cell Counting Kit-8 (CCK8) assay and flow cytometry were, respectively, utilized to detect the cell viability and apoptosis levels of 16-HBE cells. ELISA and Western blot were, respectively, used to analyze the inflammatory markers and JAK2/STAT3 signals. Immunofluorescence assay was performed to identify M0 and M2 cells. As shown in results, ZKPC perturbed the expression of IL-6 inducible genes important for apoptosis, oxidative and inflammatory response, which was enhanced by JAK2 inhibitor. Besides the inhibitory effects on the phosphorylation levels of JAK2/STAT3, ZKPC markedly increased cell viability and reduced apoptosis in human bronchial epithelial cells (16-HBE) cultured in the supernatant of M2 cells. Collectively, ZKPC could inhibit the IL-6-induced JAK/STAT3 signaling cascade, increase cell viability and decrease apoptosis induced by the supernatant of M2. A more comprehensive understanding of the action mechanism of ZKPC on JAK2/STAT3 signaling pathway in human bronchial epithelial cells induced by IL-6 or M2 supernatant will enable ZKPC development in the control of asthma.

## Introduction

Zhike Pingchuan Granule (ZKPC) is a traditional Chinese medicine (TCM) preparation developed according to clinical prescription, which was composed of platycodon grandiflorum, Schizonepeta, aster, Radix Tangeriniae, Rhizoma dioscoreae, licorice as raw materials [1]. It is mainly used for treating bronchial asthma (deficient asthma) of cough, shortness of breath, chest tightness, throat noise, shortness of breath, fatigue and sweating [2]. Clinic has confirmed that the drug shows potency in relieving cough, expectorant and asthma, partly due to its prevention against acute and chronic inflammation and regulation on humoral immunity and cellular immunity.

In clinical practice, most drugs for cough and asthma are chemical drugs with clear chemical composition and multicomponent Chinese traditional medicine. Although they can quickly and accurately remove patients’ uncomfortable symptoms, it is hard for them to cure patients’ diseases due to their simpleness in efficacy. Pingchuan Mixture has been studied to affect inflammatory cell levels and interleukin (IL)-5 levels in bronchial alveolar lavage fluid of asthma pigs. IL-6 levels showed an increase in asthma patients, which could facilitate migration of airway epithelial cell through regulating Akt/ glycogen synthase kinase 3 β signals [3–5]. IL-6 was reported to produce a pathogenic effect in an asthma model, the inactivation of which reduced eosinophilia in the lungs [6,7]. After binding to the receptor, IL-6 preferentially activates JAK and STAT3, the phosphorylated form of which enters the nucleus, regulates the transcription of downstream target genes, and participates in the regulation of inflammation. The activation of IL-6 trans-signaling was also implicated in destroying epithelial integrity and genes enrichment relating to airway remodeling [8]. JAK2/STAT3 plays a vital role in the progression of asthma [9]. JAK2 signals were implicated in regulating proliferation and promoting apoptosis of human airway smooth muscle cells [10]. In addition, JAK2-STAT3 signal was closely linked to the mediation of MUC5AC expression in IL-13-induced human bronchial epithelial cells [11].

Macrophages, as specialized airway phagocytes [12], also play an important role in airway inflammation in asthma [13,14]. They can be polarized into different phenotypes in different microenvironments and participate in the immune regulation process of the body through the change in the proportion of M1 type and M2 type, playing an important role in the pathophysiological immune escape, inflammation, tissue repair and other processes [15]. Some studies indicated that the increased number of M2 macrophages in asthma was related to the severity of asthma [12,16]. M2 polarization of macrophages is associated with the inflammatory response in asthma. Furthermore, circulating M2-like phenotype was found to present increased number in patients with bronchial asthma [17]. Further review showed that M2-type macrophage polarization may aggravate the inflammatory airway response of asthmatic patients through the expression of cytokines [18]. It was reported that M2 macrophages could produce more IL-6 levels than M0 macrophages [19]. The pathogenic role of IL-6 and ZKPC in IL-6-stimulated 16-HBE cells was not fully understood, as well as the effects of ZKPC on the M2 supernatant-induced 16-HBE cells. We hypothesized that the mechanism of ZKPC against asthma could be associated with JAK2/STAT3 pathway. Therefore, the study was designed to analyze the mechanism of ZKPC in IL-6 or M2 supernatant-stimulated 16-HBE cells and further reveal its potential mechanism involved in bronchial asthma.

## Method

### Cells

16- 1 HBE cells were cultured in DMEM medium containing 10% FBS at 37°C with 5% CO_2_. The solution was changed at intervals of 1 d and digested by trypsin every 3–4 d. The cells at logarithmic growth stage were digested and inoculated into 6-well plates. When the cells had grown to about 80% fusion, the medium was replaced with serum-free medium and then continuously utilized to culture cells for 16 h. Then, the cells were treated with IL-6 at different concentrations or pre-treated with JAK2 inhibitor Fedratinib (3 nM) or ZKPC. Different concentrations of IL-6 used in this study were based on preliminary experiment.

### CCK-8 assay

The logarithmic growth phase cells were inoculated in 96-well plates (2 × 10^7^/L). After corresponding treatment, 10 μL CCK-8 reagent was added to the cells and incubated with cells for 2 h. The absorbance of each hole at the wavelength of 450 nm was measured by a microplate reader. Three repeated holes were set for each group to take the mean value while a single hole was set with only medium added for blank control. The cell viability was calculated using the following formula: cell viability * % = [A (treatment) – A (blank)]/[A (control) – A (blank)]. ‘A’ means absorbance of each group.

### Flow cytometry

Cell apoptosis was detected using ANNEXIN V- FITC/PI kit according to the manufacturer’s protocol (Solarbio, Beijing, China). The cells were digested by trypsin without EDTA and collected by centrifugation. The cells were rinsed with PBS for 3 times, isolated and resuspended with binding buffer. Annexin V-FITC was added in the dark for incubation at room temperature for 10 min, and PI was added in the dark for another 5 min. Flow cytometry was used to detect fluorescence intensity at the excitation wavelength of 488 nm and emission wavelength of 530 nm.

### The detection of inflammatory markers and oxidative stress levels

After treatment, IL-1β, TNF-α, and IL-6 of each group were detected according to the corresponding ELISA kit in accordance with the instructions provided by Abcam (England), and reactive oxygen species (ROS), malondialdehyde (MDA) and Superoxide Dismutase (SOD) activity were measured according to the instructions provided by Nanjing Jiancheng Bioengineer Institute (Nanjing, China).

### Western blot

The total amount of protein loading was adjusted to 60 μg after quantitative analysis with BCA protein quantitative kit, and 4 times of protein loading buffer was added. 8% SDS-PAGE was used to separate proteins. Then, the protein was transferred to PVDF membrane by electrotransfer and was sealed on a shaking bed with 5% skim milk at room temperature for 90 min. Then, the primary antibody (anti-IL-6, Cat.no.ab233706; anti-Bax, Cat.no.ab182734; anti-cleaved caspase 3, Cat.no.ab32042; anti-p-JAK2, Cat.no.ab32101; anti-t-JAK2, Cat.no.ab245303; anti-CCR3, Cat.no.ab227032; anti-CCR4, ab1669; anti-p-STAT3, Cat.no.ab267373, anti-t-STAT3, Cat.no.ab68153; anti-GAPDH, Cat.no.ab9485. Abcam, England) was added and incubated with PVDF membrane at 4°C overnight. After reheating, TBST was added to wash PVDF membrane for 3 times, 5 min each. Then, PVDF membrane was incubated with secondary antibody (HRP-conjugated Goat Anti-Rabbit IgG, Cat.no.ab7090, Abcam, England) at room temperature for 90 min and washed with TBST for 3 times, 10 min each. The color was developed in the darkroom. ImageJ was used for gray level analysis.

### Induction of macrophages polarization

THP-1 cells were seeded into 6-well plates with 8*10^5^ cells/well. 100 nM PMA (Sigma) was added to the cells after incubation for 24 h to induce cells to differentiate into M0 macrophages. The cell medium was discarded, and the cells were washed with PBS for three times. DMEM medium containing no serum was added into the cells while IL-4 and IL-13 were also added to induce M0 macrophages polarization to M2 type [20].

### Immunofluorescence assay

THP-1 cells were collected and washed with PBS for three times and then fixed with 4% paraformaldehyde for 15 min. Following the use of 0.5% TritonX-100 for 20 min to increase membrane permeability, cells were washed and blocked with 5% BSA for 30 min. Then, the cells were incubated with primary antibodies at 4°C overnight, followed by incubation with secondary antibodies at room temperature for 2 h. The cells were observed and photographs taken under a fluorescent microscope (Olympus).

### Statistical analysis

Prism 6.0 statistical software was used for one-way ANOVA analysis of experimental data, followed by tukey’s test between two groups. The data were shown as mean ± standard deviation (SD). Each experiment was repeated at least three times.

## Result

### Fedratinib enhanced the effects of ZKPC on enhancing cell viability in IL-6-stimulated 16-HBE cells

**Figure 1.**
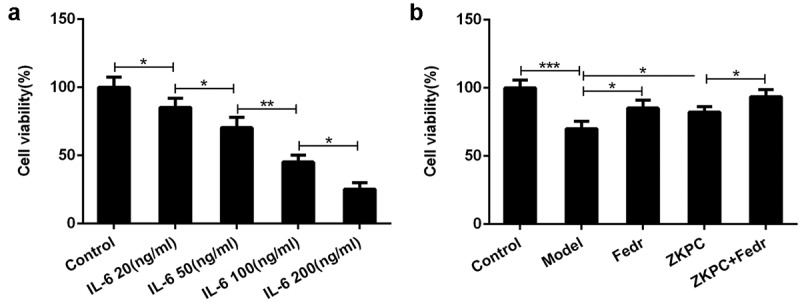
(a) IL-6 at different concentration was utilized to stimulate 16-HBE cells for 6 h. (b) ZKPC increased cell viability in IL-6-stimulated 16-HBE cells. *P < 0.05, **P < 0.01, ***P < 0.001. Model: 16-HBE cells received 50 ng/mL IL-6 treatment for 6 h. Fedr: 16-HBE cells were co-treated with IL-6 and Fedratinib. ZKPC: HBE cells were stimulated with IL-6, Fedratinib, and Zhike Pingchuan granule. Each experiment was repeated at least three times. Data was presented as mean±SD

The presentation of IL-6 increase in asthma patients represented its vital pathogenic role in asthma [3–5,7]. To determine how IL-6 affected cell viability, IL-6 at different concentrations was employed to treat 16-HBE cells for 8 h. Treatment with IL-6 significantly reduced 16-HBE cell viability in a dose-dependent manner (Figure 1(a)). 50 ng/ml of IL-6 was used to perform further experiments. 16-HBE cells were treated by ZKPC or Fedratinib (JAK2 inhibitor) for 24 h prior to treatment with IL-6. After 24 h, 16-HBE cells were induced by IL-6 for 8 h. Fedratinib treatment markedly increased cell viability compared with Model group, which implied that the inhibition of JAK2 signal significantly enhanced cell viability. Simultaneously, ZKPC treatment also notably increased cell viability compared to Model group. Most importantly, the cell viabilities were further increased by the co-treatment of ZKPC and Fedretinib, which indicated that ZKPC enhanced cell viability through JAK2 signal (Figure 1(b)).

### ZKPC significantly reduced cell apoptosis, inflammatory factor levels, and oxidative stress

**Figure 2.**
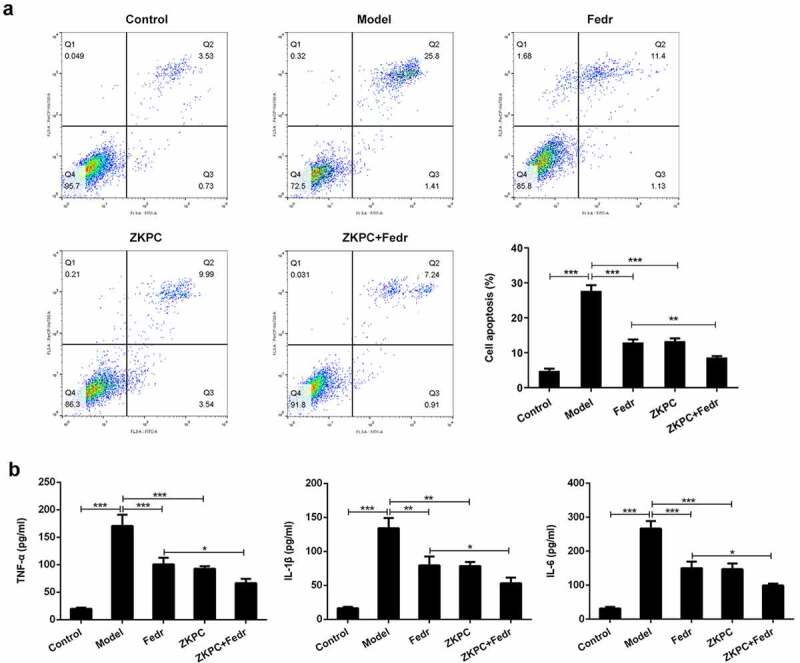
(a)-(b) The apoptosis levels were analyzed through Flow cytometry in IL-6 induction. (b) Inflammatory markers were assessed by ELISA. *P < 0.05, **P < 0.01, ***P < 0.001. Model: 16-HBE cells received 50 ng/mL IL-6 treatment for 6 h. Fedr: 16-HBE cells were co-treated with IL-6 and Fedratinib. ZKPC: HBE cells were stimulated with IL-6, Fedratinib and Zhike Pingchuan granule. Each experiment was repeated at least three times. Data was presented as mean±SD

**Figure 3.**
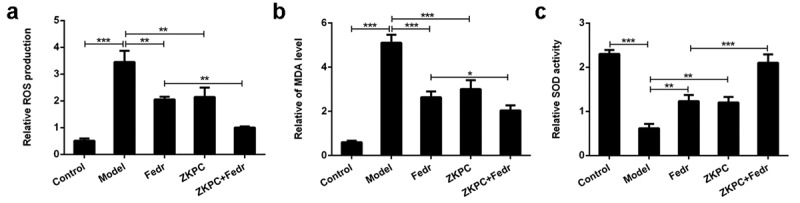
(a)-(c) We detected ROS, SOD, and MDA levels to evaluate oxidative stress through ROS, SOD, or MDA kit, respectively. *P < 0.05, **P < 0.01, ***P < 0.001. Model: 16-HBE cells received 50 ng/mL IL-6 treatment for 6 h. Fedr: 16-HBE cells were co-treated with IL-6 and Fedratinib. ZKPC: HBE cells were stimulated with IL-6, Fedratinib, and Zhike Pingchuan granule. Each experiment was repeated at least three times. Data was presented as mean±SD

To further assess the effects of ZKPC on the IL-6-induced 16-HBE cells, the apoptosis, inflammatory marker levels, and oxidative stress were determined in the bronchial epithelial cells. Fedratinib and ZKPC could reduce cell apoptosis, pro-inflammatory factor, and oxidative stress levels in 16-HBE cells induced by IL-6 (Figures 2(a-c) and 3). IL-6 treatment markedly induced apoptosis rate of 16-HBE cells relative to control group through analysis from flow cytometry, while ZKPC markedly reduced IL-6-stimulated cell apoptosis. Besides, we found that ZKPC can significantly lessen the levels of pro-inflammatory factors including TNF-α, IL-1β, and IL-6, indicating that ZKPC could ameliorate inflammatory response induced by IL-6. Then, ROS level, which was recognized as a marker of oxidative stress, was reduced upon ZKPC or Fedratinib treatment (Figure 3(a)). Moreover, as compared to the control group, MDA expression was increased and SOD activity was reduced in IL-induced 16-HBE cells (Figure 3(b,c)), suggesting that ZKPC regulated oxidative stress levels possibly through reducing MDA levels and increasing SOD activities. Simultaneously, Fedratinib treatment could markedly enhance the effects of ZKPC on oxidative stress in IL-induced 16-HBE cells.

### ZKPC regulated apoptosis-related proteins and JAK/STAT3 signals

**Figure 4.**
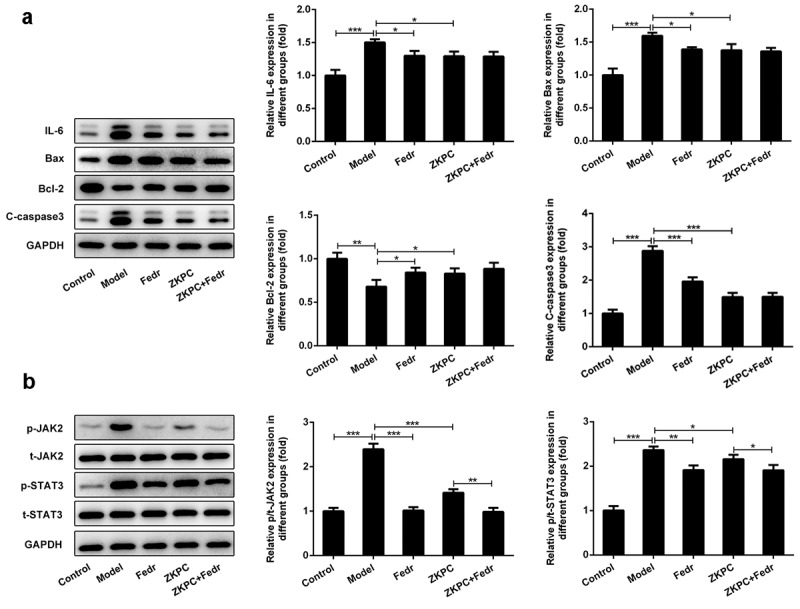
(a) ZKPC and Fedratinib regulated Bax, Bcl-2, and cleaved-caspase 3. (b) ZKPC reduced the phosphorylation levels of JAK/STAT3 signals. *P < 0.05, **P < 0.01, ***P < 0.001. Model: 16-HBE cells received 50 ng/mL IL-6 treatment for 6 h. Fedr: 16-HBE cells were co-treated with IL-6 and Fedratinib. ZKPC: HBE cells were stimulated with IL-6, Fedratinib, and Zhike Pingchuan granule. Each experiment was repeated at least three times. Data was presented as mean±SD

To determine how ZKPC suppressed apoptosis induced by IL-7, the expression of proteins involved in apoptosis were detected by Western blot. The aforementioned flow cytometry results demonstrated that ZKPC markedly reduced cell apoptosis rate through JAK2 signals in IL-6-stimulated 16-HBE cells. We found that Fedratinib and ZKPC could significantly reduce the expression of Bax and cleaved-caspase 3 and promote Bcl-2 expression relative to the Model group (Figure 4(a)). However, co-treatment with Fedratinib and ZKPC did not significantly affect Bax, cleaved-caspase 3, and Bcl-2 expression, possibly owing that the JAK2 signal was blocked by ZKPC or Fedratinib. Then, we analyzed the effects of ZKPC on JAK2/STAT3 signals. As was shown in (Figure 4(b)), the ZKPC markedly reduced the phosphorylation levels of JAK2 and STAT3 but did not notably affect the levels of t-STAT3, which indicated that ZKPC could inhibit the JAK2/STAT3 pathway. The pre-treatment of Fedratinib could further suppress JAK2 signals. The results showed that ZKPC could regulate JAK/STAT3 signals.

### ZKPC increased cell viability, decreased apoptosis, and reduced CCR3 and CCR4 expression levels

**Figure 5.**
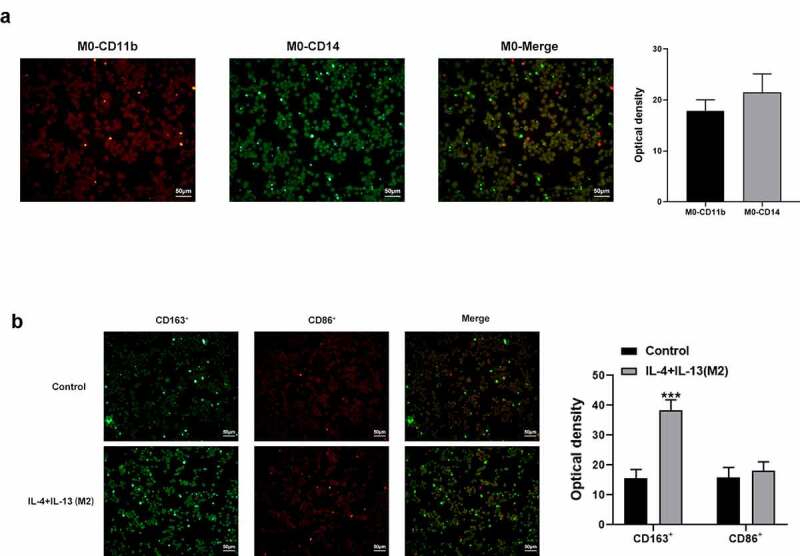
(a) PMA (10 ng/ml) was added to induce 16-HBE cells. (b) IL-4 and IL-13 were added to induce the polarization of M0 cells into M2 cells

**Figure 6.**
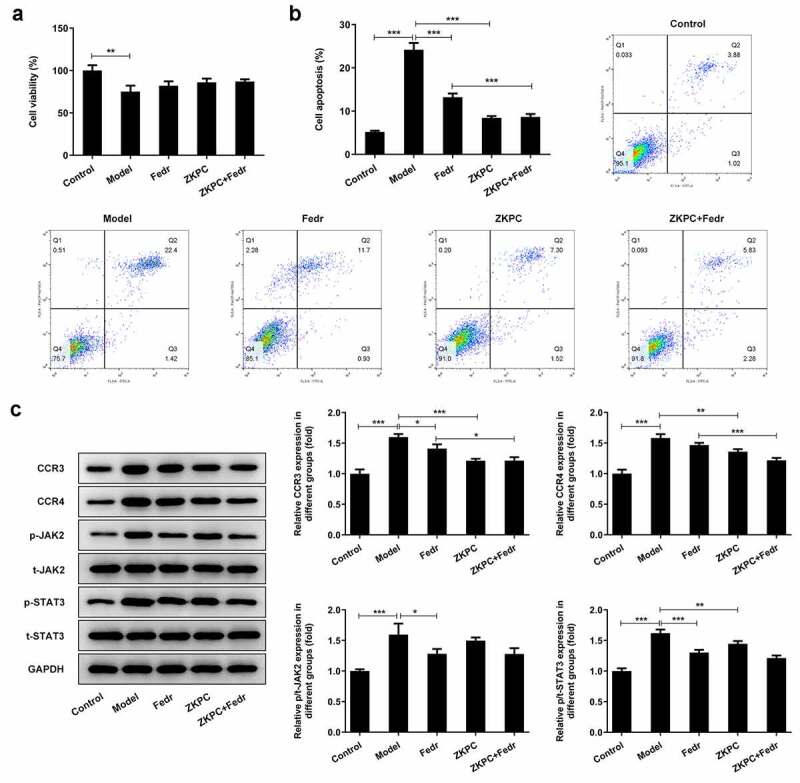
(a) ZKPC increased 16-HBE cell viability cultured in the supernatant of M2 cells. (b) ZKPC decreased 16-HBE cell apoptosis cultured in the supernatant of M2 cells. (c) ZKPC altered the expression levels of CCR3 and CCR4 and the ratio of p-STAT3/STAT3 in 16-HBE cells cultured in the supernatant of M2 cells. *P < 0.05, **P < 0.01, ***P < 0.001. Model: 16-HBE cells were treated with the supernatant of M2. Fedr: 16-HBE cells were co-treated with the supernatant of M2 and Fedratinib. ZKPC: HBE cells were stimulated with the supernatant of M2, Fedratinib, and Zhike Pingchuan granule. Each experiment was repeated at least three times. Data was presented as mean±SD

To further explore the mechanism of ZKPC in bronchial asthma, the medium culturing M2 macrophages was collected to treat 16-HBE cells. THP-1 cells were induced by PMA (10ng/ mL), and adherent cells (M0) were collected and identified through the immunofluorescence method. The results are shown in Figure 5(a). Next, as shown in Figure 5(b), IL-4 and IL-13 were used to induce M0 cells polarization into M2 cells, which were identified by the immunofluorescence method. The supernatant of M2 cells was used as the conditioned medium to culture 16-HBE cells (Model group). The cell viability was significantly decreased, and apoptosis levels were increased by the addition of the supernatant of M2 cells compared with the control group. ZKPC was added to the supernatant, which was found to increase cell viability and decrease cell apoptosis (Figure 6(a,b)). Additionally, ZKPC resulted in decreased levels of CCR3 and CCR4 compared with Model group, which showed an increase in Model group when relative to control group (Figure 6(c)). There was no significant difference between Model group and Fedratinib group. It is shown in Figure 6(c) that the ratio of JAK2 and its phosphorylation levels and the ratio of STAT3 and its phosphorylation levels were elevated in Model group compared with control group. However, the ratio of STAT3/p-STAT3 was markedly reduced by ZKPC compared with Model group, and no significant difference was found between ZKPC and Model group. These results indicated that ZKPC reduced the effect of some factors secreted by M2 macrophages on corresponding receptors possibly through JAK2/STAT3.

## Discussion

This study investigated the mechanism by which ZKPC protects against apoptosis, inflammation-associated markers, and oxidative stress in human bronchial epithelial cells treated with IL-6 or the medium of M2 macrophages. In the study, IL-6 treatment significantly reduced cell viability of human bronchial epithelioid cells in a dose-dependent manner. IL-6 plays a vital role in asthma. In asthma mice, macrophages and dendritic cells were found to be the key sources of pathogenic IL-6. Plasma IL-6 was positively correlated with clinical features of metabolic dysfunction in more severe asthma [21–23]. Knockdown of IL-6 in macrophages could markedly reduce the levels of key indicators in type 2 allergic inflammation, and the inactivation of IL-6 played a protective role in asthma mice [7].

ZKPC treatment markedly contributed to increased cell viability and decreased apoptotic levels in IL-6-treated human bronchial epithelial cells, suggesting the protective role of ZKPC against IL-6. IL-6 inhibitors were considered to ameliorate metabolic dysfunction in much severe asthma, indicating the positive correlation of IL-6 with clinical presentations of metabolic dysfunction in this disease [23]. IL-6 receptor blockade has been used to treat persistent asthma [24].

IL-6 induced the activation of JAK/STAT3 to modulate cell processes including apoptosis and proliferation [25]. Consistent with previous finding, IL-6 induced the phosphorylation of JAK2/STAT3 in our study, which implied that IL-6 could activate JAK2/STAT3 signal. Since ZKPC markedly reversed the phosphorylation levels of JAK2/STAT3 induced by IL-6 and lessened IL-6 levels relative to Model group, we concluded that ZKPC reduced cell apoptosis, inflammatory response and oxidative stress possibly through IL-6/JAK2/STAT3 pathway. JAK2/STAT3 signal has been proved to regulate cytokine levels in human bronchial epithelial cells [26]. Besides, JAK2/STAT3 was involved in regulating airway smooth muscle cell inflammatory response, proliferation, and apoptosis [9]. Our study showed that ZKPC regulated cytokines production, apoptosis, and oxidative stress levels in IL-6-stimulated 16-HBE cells. In asthma patients, there was an obvious increase in epithelium apoptosis [27]. Furthermore, regarding the mechanism of asthma pathogenesis, many experts held that it was ROS that promoted inflammation and oxidative stress, thereby mediating the pathological changes of asthma [28–30]. One of the effective components in ZCPK, Platycodon grandiflorum was reported to reduce cell apoptosis and regulate NF-κB-mediated inflammation and PI3K/Akt/apoptosis signaling pathways [31,32].

Substantial lines of evidence have indicated that M2 macrophages exhibited inextricable link to asthma. A study showed that the number of M2 macrophages was significantly higher in patients with bronchial asthma and asthmatic mice compared with healthy controls [33]. The proportion of macrophages expressing mannose receptors and transglutaminase 2 in lung biopsies of patients with asthma was also significantly increased. Specifically, the number of M2 macrophages was positively correlated with the degree of airway inflammation, as evidenced by a house dust mite-induced asthma mouse model [34]. Further, blockage of TG2, the product of M2 macrophages, significantly reduced airway hyperresponsiveness, IgE expression level and inflammatory cell infiltration, which suggested the potential of M2 macrophage inhibition in treating asthma [35]. Intriguingly, in our study, ZKPC was found to be able to resist the supernatant of M2-induced decrease in cell viability and apoptosis, and reduce the levels of chemokine receptors, CCR3, and CCR4, which are involved in lung inflammation in asthma [36,37]. ZKPC could reduce the effect of some factors secreted by M2 macrophages on corresponding receptors (but its downstream may be through the JAK pathway). The effects of ZKPC and Fedr alone in cell proliferation, apoptosis, and cytokines production is still unclear, which is the limit of this study.

## Conclusion

These data provide sufficient rationale to approve that ZKPC exerts beneficial effects on reducing cell apoptosis, inflammation, and oxidative stress and alleviating cell apoptosis induced by the medium of M2 macrophages through regulating IL-6/JAK2 /STAT3 pathway.

## Data Availability

The datasets used and/or analyzed during the current study are available from the corresponding author on reasonable request.

## Highlights

1. The effects of ZKPC on suppressing IL-6-induced apoptosis could be related to JAK2/STAT3.
2. JAK2/STAT3 could participate in the inhibition of ZKPC for oxidative stress induced by IL-6.
3. ZKPC reduces apoptosis possibly by blocking JAK2/STAT3 activation by cytokines secreted by M2.
